# South African Herbal Teas and Diabetes‐Mediated Mitochondrial Dysfunction: Emerging Evidence and Mechanistic Insights

**DOI:** 10.1111/1750-3841.71351

**Published:** 2026-08-12

**Authors:** Shanika Reddy, Naeem Sheik Abdul, Taskeen Fathima Docrat, Jeanine Marnewick

**Affiliations:** ^1^ Applied Microbial and Health Biotechnology Institute Cape Peninsula University of Technology Bellville South Africa; ^2^ Department of Biochemistry Stellenbosch University Stellenbosch South Africa

**Keywords:** diabetes, herbal teas, mitochondrial dysfunction, oxidative stress

## Abstract

Type 2 diabetes mellitus (T2DM) is a central component of the metabolic syndrome (MetS) and is associated with underlying mitochondrial dysfunction. Disruption of mitochondrial homeostasis impairs cellular energy metabolism and contributes to the overproduction of reactive oxygen species (ROS), leading to oxidative stress, chronic inflammation, and insulin resistance. These mitochondrial abnormalities exacerbate insulin resistance and impair pancreatic β‐cell function, thereby accelerating the progression of T2D. Emerging evidence highlights the therapeutic potential of plant‐derived biomolecules, particularly polyphenols found in traditional herbal teas, for mitigating oxidative stress and restoring mitochondrial integrity. This review explores the pathophysiological role of mitochondrial dysfunction and oxidative stress in T2D, with a focus on insulin action and β‐cell function. We review three South African herbal teas (Rooibos, Honeybush, and Bush tea) and summarize how, through regulation of the oxidoreductase enzyme system, upregulation of endogenous antioxidant enzymes, inhibition of pro‐oxidant enzymes, and support of mitochondrial biogenesis, demonstrating promising bioactivity. The review synthesizes current evidence on the herbal tea's key phytochemicals and their proposed mechanisms of action, highlighting their relevance in mitigating redox imbalance and preserving mitochondrial integrity within the context of diabetes. In addition, factors influencing their nutraceutical value, such as phytochemical composition, processing conditions, and bioavailability, are considered in relation to functional food development. By linking their antioxidant and anti‐inflammatory properties to mitochondrial protection, this review underscores their potential role as complementary interventions in the management of T2D. Although emerging in vitro and in vivo studies suggest promising bioactivity of the herbal teas, the current evidence base remains limited by a lack of well‐controlled human clinical trials.

**Abbreviations**
ARE: antioxidant‐response‐element
CVD: cardiovascular disease
DAMPs: damage‐associated molecular patterns
d‐galN: d‐galactosamine
ETC: electron transport chain
FRE: fermented Rooibos extract
GCLC: glutamate–cysteine ligase catalytic
GCLM: glutamate–cysteine ligase modulator
GSH: glutathione
GSS: glutathione synthetase
HbA1c: hemoglobin A1c
IL‐1β: interleukin‐1β
IL‐6: interleukin‐6
iNOS: inducible nitric oxide synthase
KEAP1: Kelch‐like ECH‐associated protein 1
MetS: metabolic syndrome
mtDNA: mitochondria DNA
mtROS: mitochondrial ROS
NFκB: nuclear factor κB
NLRP3: nucleotide‐binding and oligomerization domain‐like receptor family pyrin domain‐containing 3
NRF‐1: nuclear respiratory factor‐1
NRF2/EpRE: nuclear factor erythroid 2/electrophile‐responsive element
OxPhos: oxidative phosphorylation
P2X7r: purinergic P2X7 ionotropic receptor
Pdx‐1: insulin promoter factor 1
PGC‐1α: proliferator‐activated receptor gamma coactivator‐1 alpha
ROS: reactive oxygen species
SIRT1: sirtuin 1
SOD2: superoxide dismutase 2
T2DM: Type 2 diabetes mellitus
TCA: citric acid cycle
TFAM: mitochondrial transcription factor A
TNF‐α: tumor necrosis factor‐alpha
UCP2: uncoupling protein 2

## 1 Introduction

Metabolic syndrome (MetS) is a complex cluster of metabolic abnormalities that include hyperglycemia, abdominal obesity, hypertriglyceridemia, hypertension, and low high‐density lipoprotein cholesterol levels, which, if left untreated, are associated with cardiovascular disease and diabetes. Clinical and epidemiological evidence strongly links over‐nutrition and a sedentary lifestyle as key contributors to diabetes (Zhu et al. 2023; Cavallo et al. 2022; Zhang, Shen, et al. 2022; Ruze et al. 2023). With the advent of modern food processing and efficient supply chains, the high prevalence of diabetes, especially Type 2 diabetes (T2D), is no longer unique to the developed Western nations of North America and Europe (Liu et al. 2022; Ali et al. 2022). Socioeconomic improvements, together with expanded urbanization and nutrition transition to energy‐dense foods, have led to an increased prevalence of T2D in many developing countries on the African and Asian continents (Gassasse et al. 2017; Mattei et al. 2015; Popkin 2015).

Lifestyle interventions, with an emphasis on physical activity and healthy eating, have proven helpful for T2D patients (Amerkamp et al. 2025; Salvia and Quatromoni 2023; Barrea et al. 2023). Recent studies have highlighted the potential of alternative therapy, including apitherapy and phytotherapy, as strategies to prevent and treat T2D (Jagua‐Gualdrón et al. 2025; Governa et al. 2018). Among these studies, the emergence of herbal teas as a treatment for T2D is noteworthy (Yang et al. 2014; Li et al. 2016; Zhang, Zhang, et al. 2022). Tea is a widely consumed beverage, containing a plethora of bioactive molecules that are associated with its health benefits (Pan et al. 2016; da Silva Pinto 2013; Khan and Mukhtar 2018). Several plants have traditionally been used as a “tea” or tisane in South Africa (Watt and Breyer‐Brandwijk 1962). However, only Rooibos (*Aspalathus linearis*) and Honeybush (*Cyclopia* spp.) have attained international acclaim, whereas Bush tea, brewed from *Athrixia phylicoides* (another indigenous South African plant), is recognized as having “potential for commercialisation” (Joubert, Gelderblom, et al. 2008). These three plant species represent the established (Rooibos), developing (Honeybush), and emerging (Bush tea) markets.

The use of Rooibos and Honeybush evolved from their origins in traditional medicinal practices to widespread non‐medicinal consumption as beverages, and more recently re‐emerging within the functional foods space, where their bioactive properties are being investigated and contextualized within health care (Joubert, Gelderblom, et al. 2008), whereas the use of Bush tea for medicinal and non‐medicinal purposes is more traditional‐based and mainly used by the Sotho, Venda, Xhosa, and Zulu communities of South Africa (Lerotholi et al. 2017).

Rooibos, Honeybush, and Bush “teas” contain a unique blend of polyphenols that contribute to their acclaimed health benefits. These tisanes have shown potent antioxidant activity by scavenging ROS and activating antioxidant systems (Steenkamp et al. 2004; Von Gadow et al. 1997; Joubert, Gelderblom, et al. 2008; Joubert, Richards, et al. 2008, Gerber et al. 2015; Mcgaw et al. 2007). A growing body of scientific evidence now supports the anecdotal health claims of these three indigenous plants, which are traditionally used to treat many of the metabolic abnormalities and risk factors for developing MetS and diabetes (Beltrán‐Debón et al. 2011; Muller et al. 2011; Ajuwon et al. 2018; Lerotholi et al. 2017). Several lines of evidence have suggested, with some consistency, that mitochondrial dysfunction may be a unifying mechanism for the development of metabolic diseases and MetS (Roberts and Sindhu 2009; Masenga et al. 2023; Prasun 2020; Bhatti et al. 2017).

Mitochondria serve as metabolic hubs, integrating various bioenergetic pathways; however, their dysfunction significantly contributes to the pathogenesis of metabolic diseases, and thus, the progression of diabetes and diabetic complications, particularly in insulin‐sensitive tissues like skeletal muscle, liver, and the heart (Prasun 2020). Research supports the notion that mitochondrial dysfunction contributes to diabetes through the excessive generation of ROS and disrupted bioenergetics (Cojocaru et al. 2023; Takano et al. 2023), exacerbating insulin resistance, chronic inflammation, and cell death, increasing tissue damage in diabetic patients. These findings suggest that the dual targeting of mitochondrial function and ROS generation could be a potential therapeutic approach for addressing MetS conditions like diabetes. South African tisanes are strong candidates for dual targeting of oxidative stress and mitochondrial dysfunction due to their rich polyphenolic profiles. Key constituents, such as aspalathin and mangiferin, exhibit both direct antioxidant activity and the ability to upregulate endogenous redox defense pathways. Importantly, these compounds also modulate mitochondrial function and promote biogenesis. Given the reciprocal relationship between oxidative stress and mitochondrial dysfunction, this dual mechanistic action provides a coherent rationale for their investigation as functional interventions.

The selection of Rooibos, Honeybush, and Bush tea in this review is based on both their ethnopharmacological relevance and their increasing importance as functional food ingredients. Together, these teas offer a spectrum of evidence maturity and phytochemical diversity, allowing for the evaluation of how composition and processing influence biological function. This review examines the roles of mitochondrial dysfunction and oxidative stress in T2D, focusing on insulin action and secretion, and evaluates the potential of three South African herbal teas and their bioactive compounds.

## 2 Literature Search Strategy

Although this review does not follow a formal systematic review protocol, efforts were made to ensure a comprehensive and balanced representation of the available literature. The narrative review search was conducted using electronic databases, including PubMed, Scopus, and Google Scholar.

The search was focused on literature examining the MetS, with a focus on T2D, potential health effects of South African herbal teas, their phytochemical composition and biological activity, and their potential role in mitigating phenomena such as oxidative stress and mitochondrial dysfunction, related to T2D. Relevant studies published up to 2026 were identified using combinations of keywords such as “Rooibos,” “Honeybush,” “Bush Tea,” “South African herbal teas,” “type 2 diabetes,” “metabolic syndrome,” “mitochondrial dysfunction,” “oxidative stress,” and “bioactive compounds.”

### 2.1 Inclusion Criteria

Additional studies were identified through manual screening of reference lists of selected articles, and to enhance coverage where possible, reference lists of retrieved articles and relevant reviews were manually screened to identify additional studies. Studies were included if they (1) investigated the chemical composition or bioactive properties of Rooibos, Honeybush, or Bush herbal teas; (2) explored mechanisms associated with oxidative stress, mitochondrial homeostasis, or metabolic dysfunction; or (3) evaluated potential health effects in in vitro or in vivo models. Given the emerging nature of this field, both experimental and review articles were considered to provide mechanistic context and support comprehensive interpretation.

### 2.2 Exclusion Criteria

Studies were excluded if they were not available in English, lacked sufficient detail, or not directly relevant to the thematic scope of this review.

## 3 Polyphenols Targeting Mitochondrial Dysfunction

Polyphenols are biomolecules derived from plants that display broad antioxidant properties. The antioxidant efficiency of polyphenols is proposed to suppress ROS generation by promoting the catalytic activity and expression of antioxidant enzymes, along with the direct scavenging of ROS (Rahman et al. 2006; Truong and Jeong 2021) and by modulating the nuclear factor erythroid 2/electrophile‐responsive element (NRF2/EpRE) (Masenga et al. 2023). Other general mechanisms of action involved include inhibiting inflammasome activation and expression by decreasing NF‐кB transcriptional activity, thereby decreasing pro‐inflammatory cytokines. Polyphenols have also been shown to improve mitochondrial bioenergetics and promote mitochondrial biogenesis (Ding et al. 2024; Aryaeian et al. 2017; Cao et al. 2019).

In hyperglycemic models, although their antidiabetic effects are exemplified by (1) reducing the levels of blood glucose and hemoglobin A1c (HbA1c), (2) increasing the secretion efficiency of pancreatic β cells, and (3) thus mitigating systematic insulin resistance (Liu et al. 2019).

Clinical trials have shown that dietary supplements containing polyphenols form an indispensable part of diabetes diet therapy (Cao et al. 2019; Rienks et al. 2018). Liu et al. (2019) showed that anthocyanins and resveratrol prevent the incidence of diabetes by controlling blood glucose and HbA1c levels, thereby promoting the secretion efficiency of pancreatic β cells and ameliorating systematic insulin resistance in patients (Liu et al. 2019). Cao et al. (2019) supplemented participants (with more than one abnormal medical phenotype of MetS) with dried grape pomace, containing mainly catechins and proanthocyanidins, for 6 weeks, and revealed significant improvements in fasting insulinemia linked to Type 2 diabetes mellitus (T2DM) (Cao et al. 2019). Rienks et al. (2018) also suggested that the incorporation of dietary polyphenols, especially flavonoids, into the diet of a patient may strongly influence the improvement of T2D. Furthermore, polyphenol supplementation is well tolerated with no adverse effects, permitting future research to focus on confirming these results over longer periods and better exploring the potential mechanisms of action of the polyphenols. However, the multifaceted nature of diabetes can alter the bioavailability and bioactivity of some polyphenols, possibly increasing or negating their inherent protective effects. Collectively, these studies show the value of dietary polyphenols and their potential to mitigate T2D features and highlight how further studies are necessary to identify their underlying molecular mechanisms in diabetic patients. Finally, properly assessing selected natural teas/tisanes or related polyphenols is always advised before dubbing them novel interventions.

## 4 Evidence and Perspectives for South African Herbal Teas as Therapeutic Strategies

The available treatment modality for diabetes is essentially the control of elevated blood sugar, triglycerides, and/or blood pressure through pharmacological agents (metformin, statins, or ACE inhibitors, respectively), surgeries, and even psychotherapies (Dobrowolski et al. 2022; Fisman and Tenenbaum 2015). Although commonly practiced, these treatment strategies are not entirely efficient, as indicated by the rise in morbidity and mortality rates of patients worldwide. Additionally, many of the pharmacological agents used present several side effects, including weight gain, hypoglycemia, fluid retention, and heart failure, limiting their use (Chaudhury et al. 2017). In other cases, where lifestyle choices are known to assist in managing some MetS disease states (including diabetes), the lack of patient compliance has rendered this an inconsistent and unpredictable means of treatment (O'brien et al. 2020). Considering that a large population of lower to middle‐income earners is affected, their limited access and poor affordability of “conventional medicine,” worsen the treatment prospects of these MetS patients (Leisinger 2012; Lotfizadeh et al. 2022).

For decades, herbal tea has been consumed by millions of people around the world. Their high polyphenol and flavonoid contents, therapeutic properties, and low toxicities continue to make them attractive sources of novel therapeutic compounds. Their ease of availability, low cost, and apparent reduced side effects continue to make plant‐based preparations popular therapies, especially in rural areas, where traditional or natural alternatives are often favored. In addition to their positive attributes, several lines of evidence have associated many plant polyphenols and other related constituents with potent antioxidant properties. Consequently, there is significant interest in the antioxidant and protective effects of bioactives present in these established, developing, and emerging South African herbal teas to delay or prevent insulin resistance and disrupted insulin secretion at the onset of T2D in MetS.

Overall, the evidence base is strongest for Rooibos, which has been examined across multiple in vitro, in vivo, and limited human studies, particularly in relation to glucose uptake, insulin sensitivity, oxidative stress, and mitochondrial protection. Honeybush shows promising preclinical activity, especially for mitochondrial bioenergetics, glucose metabolism, and lipid regulation, but its evidence remains more heavily weighted toward cell and animal models. Bush tea is the least characterized of the three, with encouraging findings for glucose handling and anti‐inflammatory activity, but little direct evidence on mitochondrial mechanisms or human efficacy. Across all three teas, the most consistent biological themes are redox modulation, enhancement of endogenous antioxidant defenses, and partial restoration of mitochondrial function, although the level of mechanistic resolution and translational support differs substantially between species. A summary of previously reported antidiabetic effects is listed in Table 1 for each of tisanes reviewed in this study.

**TABLE 1:** Reported antidiabetic activity effects of three South African herbal teas.

Plant species	Antidiabetic bioassays	Organism or cell line tested	Summary
*Aspalathus linearis*	Insulin sensitivity and blood glucose regulation, anti‐inflammatory activity, and/or lipid metabolism regulation	C2C12 myotubes, Chang liver cells, rats, and human clinical trial(s)	The hypoglycemic potential of Rooibos is linked to increased GLUT4 translocation to plasma membrane via AMPK activation (Kamakura et al. 2015). Aspalathin and other flavonoids were also shown to aid in the regulation of blood glucose levels by improving insulin sensitivity and glucose uptake in muscle cells. In STZ‐induced diabetic rats, supplemented with Rooibos tea, alpha‐glucosidase inhibition, and suppression of fasting plasma glucose levels were observed (Muller et al. 2012). Rooibos and its polyphenols (aspalathin, quercetin, luteolin, etc.) neutralized experimentally induced oxidative stress both in vitro and in vivo. In turn, the reduction of oxidative stress restored and protected pancreatic β‐cells for regular insulin activity (Ajuwon et al. 2018). Rooibos also modulates inflammatory markers, IL‐6 and IL‐10, without inhibiting the acute glucocorticoid response to mild stressors in vivo, highlighting its ability to promote anti‐inflammatory states (Smith and Swart 2016). In lipid related studies, Rooibos helps lower LDL and increase HDL levels, while reducing blood pressure, supporting better vascular health, and protecting against diabetes induced heart disease (Dludla et al. 2017; Persson et al. 2010). Human studies suggest potential benefits in improving glucose tolerance (Marnewick et al. 2011)
*Cyclopia* sp.	In vitro glucose uptake and metabolism, anti‐obesity effects through oil‐red‐o staining, and glycerol release	3T3‐L1 adipocytes, C2C12 myotubes, mice, and rats	Honeybush polyphenols, such as mangiferin and hesperidin, have been reported to lower blood sugar levels by stimulating insulin secretion and promoting the regeneration of damaged pancreatic cells, activating the AMPK pathway, enhancing glucose uptake in muscle cells, and improving energy metabolism (Ajuwon et al. 2018). Various extracts of *Cyclopia* spp. have demonstrated significant inhibition potential against the α‐amylase and pancreatic lipase activity in a concentration‐dependent manner, as well as antioxidant scavenging activity in a concentration‐dependent manner (Muller et al. 2011). The organic fraction of Honeybush, with higher phenolic content than its aqueous fraction, decreased lipid content compared to its aqueous fraction in 3T3‐L1 adipocytes. In Leprdb/db mice, the organic fraction of Honeybush decreased body weight gain, without affecting food or water consumption. Furthermore, Honeybush polyphenols aid in free radical scavenging, mitigating against OS‐induced insulin resistance (Jack et al. 2017; Jack et al. 2017). In vitro studies using C2C12 myotube cells suggested the role of Honeybush in managing inflammation directly associated to diabetes (Malongane et al. 2022). Studies have also shown the promise of aqueous Honeybush extracts toward lowering cholesterol and triglyceride levels, reducing the risk of metabolic syndrome (MetS) and obesity associated with diabetes (Muller et al. 2011). Preliminary studies indicate its potential in modulating blood glucose levels, warranting further investigation
*Athrixia phylicoides*	In vitro glucose uptake and metabolism, ^14^C glucose oxidation to ^14^CO_2_, glycogen storage, anti‐inflammatory activity, and/or lipid metabolism regulation	C2C12 myotubes, Chang liver cells, 3T3‐L1, and rats	Hot water extracts of Bush tea enhanced glucose uptake in various cell lines, suggesting improved glucose utilization in insulin‐responsive tissues. While only Chang liver cells showed increased glycogen storage relative to the vehicle control used (Chellan et al. 2012). In a diet‐induced MetS rat model, Bush tea infusion was found to improve glucose homeostasis, adipokine balance, and lipid parameters, targeting elements of both type 2 diabetes and obesity in MetS and validating its ethnopharmacological use (Mokwena et al. 2021). Bush tea has shown significant in vitro and ex vivo antioxidant and anti‐inflammatory properties, linked to the mitigation oxidative stress and managing inflammation linked to diabetes, respectively (Chellan et al. 2012; Malongane et al. 2022). No clinical trials using Bush tea in diabetic studies published to date

Abbreviations: IL‐1β, interleukin‐1β; IL‐6, interleukin‐6.

With a growing interest in functional foods, research into plant‐derived bioactive compounds, particularly polyphenols, has intensified, owing to their demonstrated roles of modulating oxidative stress, inflammation, and metabolic dysfunction (Ashaolu and Adeyeye 2024). Resulting from this notion, herbal teas are increasingly recognized as effective ingestion of these bioactives, owing to their palatability, general compatibility with daily diets, and upstream potential for formulation into value‐added products. The biological efficacy of these plant‐derived compounds is not solely dependent on their presence, but also on their stability, bioavailability, metabolic transformation, and interaction with food matrices (Rein et al. 2013).

These compounds must ideally survive the acidic, harsh stomach environment and digestive processes, cross the intestinal wall, and enter the bloodstream to reach target tissues. These circumstances are shaped by the inherent physico‐chemical properties of the compounds, as well as processing and preparation conditions of herbal teas. In this context, South African herbal teas, such as Rooibos, Honeybush, and Bush tea, represent promising candidates for functional food development due to their unique phytochemical composition and favorable safety profiles.

From a food science perspective, the relevance of these indigenous brews extends beyond their biological activity to the way their phytochemical profiles are shaped by processing, brewing, and formulation. Their functionality of Rooibos and Honeybush as beverages or ingredients depends on the retention, extraction, and stability of key polyphenols, such as aspalathin, mangiferin, and rutin, which, in turn, influence their bioactivity. Consequently, variables, such as fermentation status, infusion temperature, and steeping time, can be considered as determinants of product quality (Damiani et al. 2019; Joubert and De Beer 2012; de Beer et al. 2024). Studies on the influence of Bush tea processing are lacking when compared to Rooibos and Honeybush, with investigations mainly focused on horticulture as a means of regulating the phytochemical profile (Rumani et al. 2024; Nchabeleng et al. 2012). This makes standardization of preparation and processing essential for translating these teas into reproducible functional food and nutraceutical products.

The polyphenolic fingerprint of Rooibos, Honeybush, and Bush herbal extracts is well characterized using different methods as validation points, with high quantities of aspalathin and mangiferin found in Rooibos and Honeybush, respectively (Lerotholi et al. 2017; McKay and Blumberg 2007; Arries et al. 2017; De Beer et al. 2021; Joubert, Manley, et al. 2008; Joubert, Richards, et al. 2008). The unique blend of polyphenols present in Rooibos, Honeybush, and Bush herbal extracts was shown to protect against oxidative stress, inflammation, and mitochondrial dysfunction (Dludla et al. 2020, Jack et al. 2017; Mokwena et al. 2021; Lerotholi et al. 2017), making them promising interventions for several common features of diabetes. Table 2 summarizes the unique and most abundant polyphenols across each of the three tisanes.

**TABLE 2:** A summary of the most common polyphenols in each herbal tisane.

Species	Polyphenols	References
*Aspalathus linearis*	Aspalathin^a^ Nothofagin^a^ Orientin Isoorientin Vitexin Isovitexin Luteolin Luteolin‐7‐*O*‐glucoside Chrysoeriol Quercetin Isoquercitrin Hyperoside Rutin	Stander et al. (2017)
*Cyclopia* spp.	Hesperidin^a^ Mangiferin^a^ Isomangiferin^a^ IDG (3‐β‐d‐glucopyranosyl‐4‐*O*‐β‐d‐glucopyranosyliriflophenone) IMG (3‐β‐d‐glucopyranosyliriflophenone) 4‐coumaric acid (+)‐pinitol Vicenin‐2 Eriocitrin Neoponcirin	De Beer et al. (2021), Ntlhokwe et al. (2018)
*Athrixia phylicoides*	Rutin 4‐Hydroxyphenyl propyl coumarate Chlorogenic acid 6‐Hydroxyluteolin‐7‐*O*‐β‐glucoside Quercetagetin‐7‐*O*‐glucoside Caffeic acid Quercetin Inositol Kaempferol Hymenoxin Apigenin Oleanolic acid	Reichelt et al. (2012), Lerotholi et al. (2017)

^a^Polyphenolic compound(s) unique to each listed plant species.

Different polyphenols have different functional groups that allow selective binding and modulation of key enzymes and signaling proteins across metabolic tissues. In the case of Rooibos, aspalathin possesses a dihydrochalcone (glucoside) core structure and *c*‐glucosylated dihydrochalcone with hydroxyl key functional group (Stander et al. 2017; Chaudhary et al. 2021). Similarly, Honeybush's xanthone and flavones, like mangiferin and hesperidin, have *c*‐glucosyl xanthone with phenolic hydroxyl groups and flavanone glycoside with methoxy and hydroxyls (Hering et al. 2021). Bush tea contains a high level of the quercetin‐3‐*O*‐rutinoside, rutin, and flavanol glycosides with catechol‐type hydroxyls as its key functional group (Malongane et al. 2022). All four secondary compounds possess multiple hydroxyl groups, enabling ROS scavenging and, therefore, antioxidant action, enhanced hydrogen bonding with enzymes and receptors, chelation of metal ions (preventing Fenton‐type reactions), and additional modulation of signal transduction pathways (Shen et al. 2022). From a translational and nutraceutical perspective, the diverse and complementary polyphenolic profiles of these herbal teas potentiate their synergistic phytochemical matrices, which may enhance overall biological efficacies. In turn, this also presents them as promising adjuncts in nutraceutical strategies for diabetes and other MetS pathologies.

As previously mentioned, processing methods, during farming, handling, and storing, play a critical role in determining the final phytochemical content and functional properties of these herbal teas. Evidence shows how processing directly influences phenolic stability and attributes of compounds, as with the traditional fermentation processing of Rooibos, where the dihydrochalcone found in Rooibos (aspalathin) is highly susceptible to oxidative degradation and heat degradation. As the process targets its key compounds, there is reduced antioxidant capacity compared to its unfermented counterpart (green Rooibos) (Villaño et al. 2010).

Similarly, Honeybush tea is primarily consumed in its fermented form; however, its compounds like mangiferin are more stable, and notably less affected by processing and oxidation than the compounds in Rooibos tea. The unique tricyclic xanthone backbone combined with multiple aromatic hydroxyl groups is reported to allow for its acclaimed stability against processing. Minimally handled and processed, green varieties also exist for consumers seeking higher antioxidant profiles. Unlike Rooibos and Honeybush teas, limited research exists on how the processing of Bush tea affects its respective compounds; however based on its phenolic profile, it can be assumed that oxidation would also affect certain compounds listed.

Preparation techniques, such as infusion time, temperature, and solvent composition, directly affect the extraction efficiency and bioavailability of phytochemicals (Cacace and Mazza 2003; Herrero et al. 2006; Nkhili et al. 2009). Careful heating promotes solubility and accelerates the diffusion rate of molecules, while breaking down cell walls to release more phytochemicals, while optimized steeping time permits adequate time for the solvent to penetrate the plant matrix and extract target compounds. Finally, solvent composition and solvent ratios ensure that the mixture does not become saturated. In the case of the teas, changes to their specific optimized parameters (steeped for too long, at elevated temperatures, or with incorrect solvents and solvent ratios) can lead to significant differences in polyphenol yield and antioxidant capacity and poorly affect the functional efficacy of the final beverage (Abd Alelah et al. 2026; Shahidi and Pan 2022). Furthermore, suboptimal extraction may limit the availability of key bioactives required to exert protective effects on mitochondrial function, whereas optimized conditions can enhance polyphenol yield and antioxidant capacity.

Finally, post‐handling, processing, and extraction, the interaction of bioactive compounds with other dietary components, such as proteins or lipids, can further influence stability and absorption. Food processing processes may disrupt the activity of phytochemicals or reduce bioaccessibility. For example, proteins act as both protectors and competitors for bioactive compounds; where proteins, like casein and whey, can bind to polyphenols, protect and carry them through the harsh, acidic environment of the stomach, or bind too strongly, prevent absorption, and render the bioactive useless. Similarly, lipids can form micelles around the bioactives, aiding in the delivery and absorption or oversaturate the bioactive compound requiring enzymes to assist in their digestion and bioabsorption (Rashidinejad et al. 2021).

Therefore, standardization of both processing and preparation conditions is essential when evaluating the health‐promoting properties of herbal teas. A thorough understanding of food matrix dynamics is equally critical, as it underpins the design of functional foods and the optimization of dietary strategies for maximal nutritional benefit, while ensuring that findings can be translated into reproducible functional food products. Collectively, these considerations underscore the importance of optimizing processing parameters to preserve bioactive compounds, which remains a key priority in the development and application of functional foods. In this context, phytochemical fingerprinting is not only useful for chemical characterization, but also for quality control, batch‐to‐batch standardization, and the development of tea‐based functional products with predictable biological activity.

For Bush tea, the limited processing literature presents a food science gap, because without defined processing effects, it is difficult to standardize quality or predict functional performance. These considerations are central to product development, as consumer‐relevant attributes such as infusion conditions, shelf stability, and reproducible phytochemical delivery determine whether a tea can credibly be positioned as a functional food.

A comparative consideration for the phytochemical profiles shows both overlap and divergence among the three teas. Rooibos is distinguished by aspalathin and related *c*‐glucosylated flavonoids, Honeybush by mangiferin and other xanthones/flavanones, and Bush tea by rutin, quercetin, and related flavonols. Although these compounds differ structurally, they converge on similar biological endpoints, including antioxidant defense, metabolic regulation, and suppression of inflammatory signaling. This suggests that the teas may act through partly shared but nonidentical mechanisms, with Rooibos appearing more strongly linked to glucose homeostasis, Honeybush to mitochondrial protection and energy metabolism, and Bush tea to anti‐inflammatory and vascular effects.

## 5 Mitochondrial Dysfunction Contributes to Diabetes and South African Herbal Teas as Interventions

### 5.1 Dysfunctional Mitochondrial Bioenergetics and Biogenesis in the Pathology of Diabetes

**FIGURE 1:**
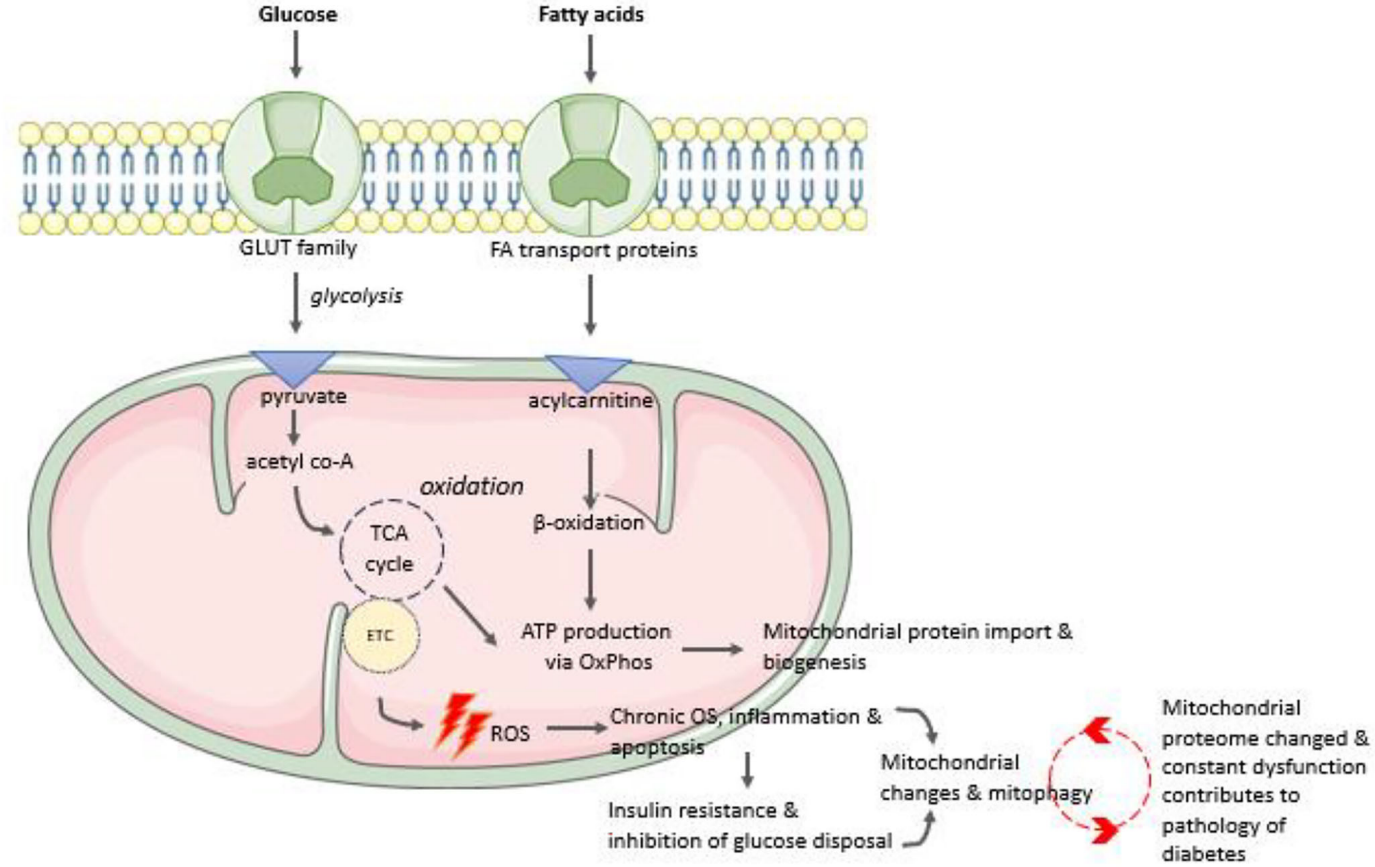
Dietary sources, such as glucose and fatty acids, undergo oxidation within the mitochondria through the citric acid cycle (TCA) and β‐oxidation. Following oxidation, there is ATP production via OxPhos, allowing mitochondrial protein import and biogenesis to support normal cell functions. The electron transport chain (ETC) is associated with mitochondrial oxidative capacity and yields the byproduct, ROS. Controlled ROS aids in cell signaling, whereas excessive ROS leads to mitochondrial changes and mitophagy. Other adverse downstream effects include inflammation, apoptosis, and insulin resistance. OxPhos, oxidative phosphorylation.

Mitochondria produce the bulk of cellular energy by integrating several metabolic processes (Cheng and Ristow 2013; Spinelli and Haigis 2018). Glucose and fatty acids obtained from dietary sources undergo oxidation within the mitochondria through the citric acid cycle (TCA) and β‐oxidation (Figure 1). The energy stored in the chemical bonds of these molecules is released during these processes as high‐energy electrons that are essential to produce ATP via oxidative phosphorylation (OxPhos) (Prasun 2020).

The decreased production of ATP by the mitochondria is an indication of mitochondrial dysfunction, often characterized by disruptions in enzyme reactions, input of nutrient signals, or oxidative respiration (Bhatti et al. 2017; Prasun 2020). There is significant evidence linking mitochondrial dysfunction with insulin resistance (Cojocaru et al. 2023; Blake and Trounce 2014). This relationship is significant given that mitochondrial dysfunction impairs both glucose‐stimulated insulin secretion by pancreatic cells and insulin‐stimulated glucose use in metabolically active organs (Kim et al. 2008).

The impact of mitochondrial dysfunction on insulin signaling is further supported by gene analyses, which show decreased expression of genes involved in mitochondrial ATP production in individuals with insulin resistance and T2DM (Mootha et al. 2003; Patti et al. 2003; Sangwung et al. 2020; Olsson et al. 2011). The consequent inhibition of glucose disposal causes significant changes in the expression profiles of many cellular proteins involved in various functions (Aluksanasuwan et al. 2017), including those of the mitochondrial proteome through posttranslational modifications of functional proteins, which may be involved in mitochondrial dysfunction during diabetes (Aluksanasuwan et al. 2020; Ma et al. 2016).

**FIGURE 2:**
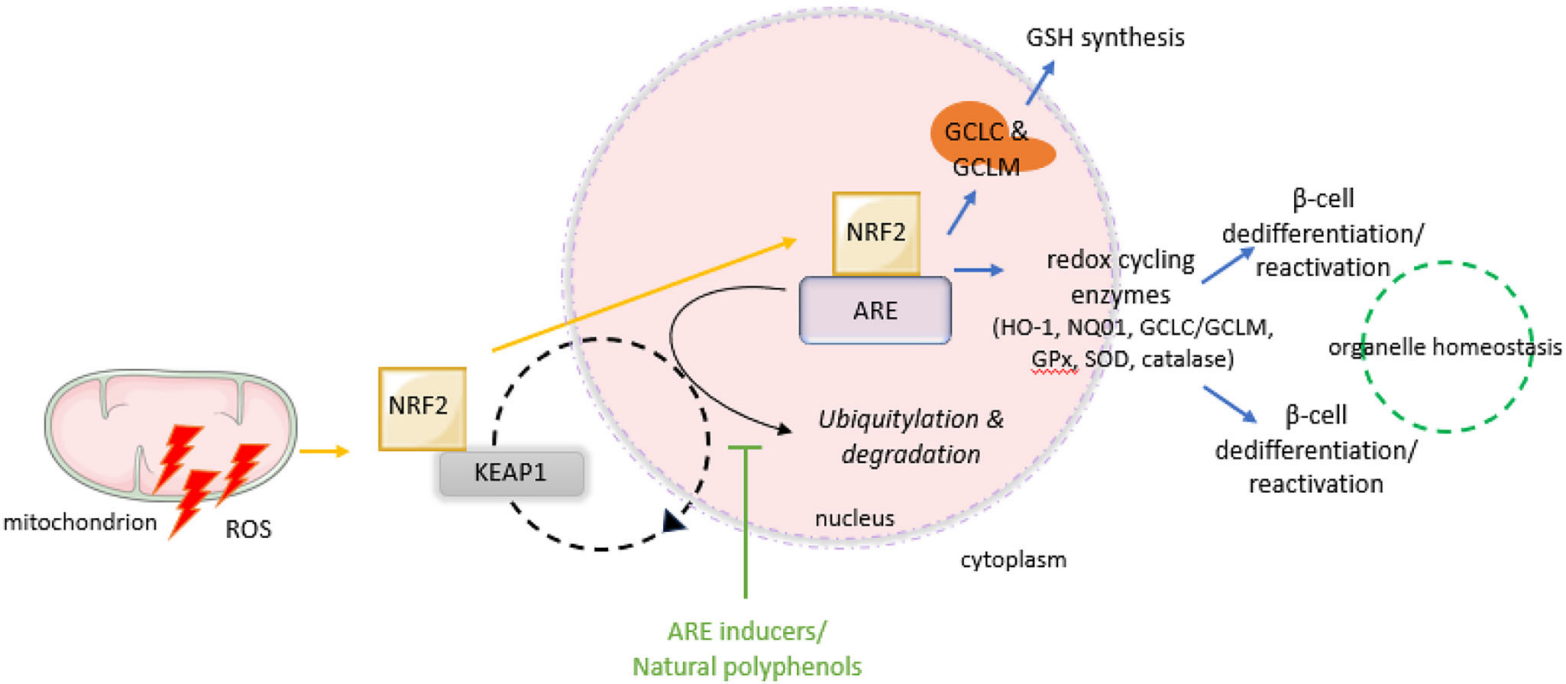
NRF2 translocates to the nucleus, where it directs various transcriptional programs in response to ROS. Endogenous NRF2 is sequestered in the cytoplasm by KEAP1 and is targeted for degradation in non‐stressed cells. Mitochondrial stress and dysfunction increase NRF2 activation and accumulation and target glutamate–cysteine ligase catalytic (GCLC) and modulator (GCLM) subunits for glutathione (GSH) synthesis. ARE inducers and natural polyphenols suppress the ubiquitylation and degradation of NRF2, permitting further NRF2‐ARE action and reducing oxidative stress. NRF2 activation of β‐cell dedifferentiation and reactivation increases β‐cell proliferation and mass and contributes to maintaining organelle homeostasis, improving overall glucose tolerance. ARE, antioxidant‐response‐element; KEAP1, Kelch‐like ECH‐associated protein 1; SOD, superoxide dismutase.

A delicate balance between nuclear and mitochondrial gene expression directs the assembly of the electron transport chain (ETC), which is closely associated with enhanced mitochondrial oxidative capacity by increasing mitochondrial number (Jornayvaz and Shulman 2010). The peroxisome proliferator‐activated receptor gamma coactivator‐1 alpha (PGC‐1α) is an integrator of the transcriptional network regulating mitochondrial biogenesis (Figure 2). PGC‐1α has been shown to facilitate downstream transcriptional regulatory circuits, including the nuclear respiratory factor‐1 (NRF‐1). NRF‐1 regulates several mitochondrial genes, such as those involved in OxPhos and mitochondrial transcription factor A (TFAM), to induce mitochondrial gene transcription (Wu et al. 2016). Evidence supports the link between insulin resistance and decreased expression of PGC‐1α‐NRF‐dependent transcription of genes involved in OxPhos, potentially affecting mitochondrial mass and substrate oxidation in diabetes (Patti et al. 2003).

Additionally, PGC‐1α is integral to normal insulin signaling and secretion, with models showing that PGC‐1α deficiency or knock out is susceptible to insulin resistance (Pagel‐Langenickel et al. 2008; Kleiner et al. 2012). A study by Oropeza et al. (2015) identified PGC‐1α as a key player in fatty acid‐mediated glucose‐stimulated insulin release and in preserving the expression of important lipolytic enzymes linked to insulin secretion within mature β‐cells (Oropeza et al. 2015). Moreover, the loss of subsarcolemmal mitochondria and associated reduction of electron transport activity have been demonstrated in insulin resistance, further substantiating the relationship between mitochondrial electron transport activity and mitochondrial density (Ritov et al. 2005). These findings support the notion that abnormal mitochondrial function in insulin secretion and activity is mainly due to reduced mitochondrial density, which may compromise the capacity of the cell to meet the high energetic demands associated with glucose sensing and insulin signaling. From this, a reduction in mitochondrial density would limit ATP availability, disrupt redox balance, and impair the coupling between glucose uptake and insulin release, signifying the importance of preserving and/or restoring mitochondrial integrity activity in T2D and MetS patients.

Rooibos and Honeybush may help mitigate some of the adverse effects of glucotoxicity on mitochondrial function. A fermented Rooibos extract (FRE) improved mitochondrial bioenergetics of cardiomyocytes exposed to high glucose by efficiently enhancing oxygen consumption, ATP production, and the maximal respiration rate. The observed effects are linked to enhanced levels of intracellular coenzyme Q9—a major component of the ETC that is often depleted in diabetics (Dludla et al. 2020). A study by Mthembu et al. (2021) reported on the ameliorative effects of aspalathin, iso‐orientin, and orientin against mitochondrial bioenergetics in cultured skeletal muscle cells. Their findings showed that the expression of genes involved in maintaining mitochondrial functions, as UCP 2, Complexes 1, and 3, were upregulated. Additionally, Hattingh et al. (2019) have shown that Rooibos treatment restored the mitochondrial membrane potential (MMP), demonstrating the potential of Rooibos to rescue mitochondrial function (Hattingh et al. 2019). The key functional hydroxyl group of aspalathin, in addition to other polyphenols in Rooibos, activates the NRF2/Kelch‐like ECH‐associated protein 1 (KEAP1) pathway and protects against glucotoxicity‐induced oxidative stress, preserving insulin production (Abdul and Marnewick 2022; Moens et al. 2020; Moens et al. 2022). This redox modulation also aids in the reduction of lipid peroxidation and increased metabolic enzyme efficiency in hepatocytes and adipocytes.

The flavonoids aspalathin, iso‐orientin, and orientin were also shown to support mitochondrial biogenesis by increasing the expression of NRF1, TFAM, and sirtuin 1 (SIRT1) (Mthembu et al. 2021), whereas a separate study showed that iso‐orientin could further enhance the expression of PGC‐1α (Luan et al. 2018). SIRT1 is thought to facilitate mitochondrial biogenesis through deacetylation and activation of PGC‐1α. These findings provide strong evidence that the flavonoids found in Rooibos have the potential to protect against mitochondrial dysfunction, which is a key hallmark in T2D.

Hot water and ethanol extracts prepared from Honeybush were shown to increase ATP production, improve mitochondrial respiration, and rescue MMP in a neuronal oxidative stress model (Agapouda et al. 2020). Studies conducted on mangiferin, the major xanthone in Honeybush support a mechanism associated with improved mitochondrial bioenergetics and biogenesis (Liu et al. 2018; Rahman and Kim 2020; Chen et al. 2021). This compound was shown to increase the glycolytic flux and enzymes involved in glycolysis, resulting in increased pyruvate and accelerated TCA cycle activity resulting from elevated expression of IDH, αKGDH, SDH, CoxI, Cyt‐c, COXIV, and ATP5g1, which resulted in an increase in TCA cycle metabolites, such as α‐ketoglutarate and fumarate. Mangiferin was also shown to increase succinate clearance by enhancing the expression and activity of succinate dehydrogenase, leading to increased ATP production. At the transcriptional level, mangiferin supports mitochondrial biogenesis by inducing the expression of TFAM, NRF1, and PGC‐1α, which correlated positively to higher levels of mitochondrial DNA (mtDNA) (Liu et al. 2018). Furthermore, *c*‐glucosylation of both mangiferin and aspalathin has been reported to offer improved stability and mitochondrial ROS (mtROS) scavenging by Honeybush and Rooibos, specifically (Fomenka and Chi 2016; Bednarska et al. 2022).

Taken together, the available evidence suggests different degrees of mechanistic validation. Rooibos has the most direct support for improving mitochondrial bioenergetics under glucotoxic stress, including effects on respiration, ATP production, membrane potential, and the expression of biogenesis‐related genes. Honeybush also shows strong mitochondrial promise, particularly through mangiferin‐mediated support of glycolytic flux, TCA cycle activity, and mitochondrial biogenesis. By contrast, Bush tea has not yet been adequately tested for direct mitochondrial bioenergetic effects, leaving an important evidence gap. Thus, although all three teas are biologically plausible mitochondrial modulators, the current data support Rooibos and Honeybush more strongly than Bush tea in this specific mechanistic domain.

### 5.2 Mitochondrial Oxidative Stress and Diabetes

Increased oxidative stress plays a complex and multifactorial role in diabetes, with patients exhibiting increased levels of biomarkers associated with oxidative damage and decreased antioxidant capacity (Martín‐Gallán et al. 2003; Bigagli and Lodovici 2019). Hyperglycemia and T2D are strongly associated with mitochondrial dysfunction and consequent oxidative stress. Mitochondria, under hyperglycemic conditions, produce large amounts of ROS, which disrupt cellular homeostasis and damage macromolecules (Szendroedi et al. 2012). The increased production of ROS further perpetuates hyperglycemia and insulin resistance by impeding the translocation of glucose transporters from the cytoplasm to the cell membrane (Fernandes et al. 2011; Leguisamo et al. 2012; Rudich et al. 1998).

Overproduction of ROS depletes the antioxidant system and consequently leads to oxidative stress in hyperglycemic environments (Caturano et al. 2023; Ghasemi‐Dehnoo et al. 2020). Oxidative stress induces alterations in mitochondrial morphology and function and causes functional abnormalities in macromolecules such as lipids, proteins, and nucleic acids (Szendroedi et al. 2012). Mitochondrial‐derived ROS and loss of ATP production under supraphysiological glucose levels are linked to β‐cell dysfunction (Brownlee 2003). This link is supported by ROS activation of uncoupling protein 2 (UCP2), which decreases the ATP/ADP ratio and thus reduces the insulin secretory response (Brownlee 2003), whereas ROS inhibits insulin gene expression at the transcriptional level by repressing insulin promoter factor 1 (Pdx‐1) and MafA transcription factor (Harmon et al. 2009).

The chemical characterization of Rooibos and Honeybush has directly led to the identification of several potential antioxidant molecules as part of their unique polyphenolic blend. Thus, significant data show that the antioxidant effects of these two herbal teas are likely to be beneficial in the hyperglycemia‐induced oxidative stress observed in the diabetic state (Ajuwon et al. 2018). However, studies showing the potential of Rooibos and Honeybush to modulate mtROS under glucotoxic conditions are less well‐characterized. The potential of Rooibos and its unique polyphenolic constituent, aspalathin, to directly detoxify mtROS under glucotoxic conditions has been linked to the enhanced expression of superoxide dismutase 2 (SOD2) in treated cells (Dludla et al. 2017; Abdul and Marnewick 2022). This antioxidant enzyme is a primary defense against mitochondrial oxidative stress, and its deletion impairs glucose‐stimulated insulin secretion and β‐cell function (Kang et al. 2014). Although no studies provide evidence for the effects of Honeybush on SOD2 levels in diabetes, several studies have implicated the potential up‐regulation of SOD2 by mangiferin, another polyphenolic constituent present in Honeybush, in different oxidative stress models (Saha et al. 2016; Huang et al. 2020; Sadhukhan et al. 2018).

The effect of Bush tea on the mitochondrial AO system under diabetic conditions remains uncharacterized, representing a notable gap given that several of its phytochemicals (Table 2) are recognized modulators of mitochondrial responses. For example, chlorogenic acid improved gene expression of SOD2 in diabetic mice (Yan et al. 2022). A study on quercetin showed that islet β‐cells were protected from oxidative stress‐induced apoptosis through upregulation of sirtuin 3 (SIRT3) and SOD2 in T2D (Wang et al. 2021). SOD2 is acetylated at lysine 68, a posttranslational modification that suppresses its enzymatic activity. The mitochondrial deacetylase SIRT3 directly binds to SOD2, catalyzes its deacetylation, and enhances its antioxidant function (Chen et al. 2011).

These findings suggest that phytochemicals in Bush tea may work synergistically to not only increase SOD2 expression but also potentiate its functional capacity, underscoring the therapeutic relevance of exploring its effects on mitochondrial AO pathways.

Accumulating evidence has shown that mtROS can activate NRF2 and induce the expression of antioxidant genes, including SOD2 (Kasai et al. 2020). The NRF2 transcription factor directs various transcriptional programs in response to oxidative stress and is considered the master regulator of the antioxidant response (Figure 2). Transcriptional activity of NRF2 is tightly regulated to assist with processes like NADPH regeneration, ROS detoxification, glutathione (GSH) and thioredoxin synthesis, and heme metabolism (Lin et al. 2016). Under basal conditions, NRF2 is inactive and sequestered by a cysteine‐rich repressor protein, Kelch‐like KEAP1. Under oxidative stress, mtROS oxidizes the cysteine bonds of KEAP1, inducing conformational changes that liberate NRF2. Free NRF2 translocates into the nucleus, where it can heterodimerize with specific proteins that encourage its binding to specific DNA sequences, such as the antioxidant‐response‐element (ARE). NRF2‐induced expression of ARE‐containing cytoprotective genes in response to cell stress includes general antioxidant pathway enzymes like reduced glutathione production, utilization, and regeneration. For GSH synthesis, NRF2 targets glutamate–cysteine ligase catalytic (GCLC) and modulator (GCLM) subunits, together with glutathione synthetase (GSS). NRF2 also targets redox cycling enzymes necessary for mediating the elimination of ROS, such as thioredoxin, thioredoxin reductase, sulfiredoxin, peroxiredoxin, glutathione peroxidase, SODs 1 and 2, catalase, heme oxygenase 1, and several glutathione *S*‐transferases. On an enzyme level, both Rooibos and Honeybush initiate detoxification of ROS by increasing the expression of catalase and levels of reduced GSH, which can be considered the first line of defense against oxygen‐free radicals (Abdul and Marnewick 2022; Van Der Merwe et al. 2017). Together these cytoprotective proteins are essential for protection against a variety of toxic and oxidative insults and in tune with many diseases associated with oxidative stress as underlying pathological features.

Studies have linked the antioxidant effects of Rooibos and Honeybush and their associated polyphenols to the stimulation of NRF2 activity, promoting their use in targeting oxidative stress induced pathologies (Van Der Merwe et al. 2017; Abdul and Marnewick 2022). Bush tea has demonstrated potential benefits in diabetic models (Mathivha et al. 2019; Chellan et al. 2012); however, its direct impact on the NRF2 antioxidant signaling pathway remains uncharacterized. Notably, several of its phytochemical constituents are known to enhance cellular defense mechanisms through activation and regulation of NRF2 (Ansari et al. 2022; Tian et al. 2016; Bao et al. 2018), suggesting a plausible, yet underexplored, mechanistic basis for its protective effects.

Cellular redox‐responsive genes, especially NRF2, play a key role in the reactivation and preservation of pancreatic β‐cells within the islets of Langerhans (Baumel‐Alterzon et al. 2021). As previously highlighted, NRF2 orchestrates a defense mechanism against oxidative stress. As β‐cells have relatively low antioxidant defenses, they are vulnerable to oxidative damage and susceptible to dysfunction, dedifferentiation, or apoptosis under oxidative stress (Khin et al. 2023). The role of redox‐responsive genes, especially NRF2, in β‐cell reactivation is a rapidly evolving area in diabetes and regenerative research.

Pancreatic β‐cells undergo progressive functional decline and eventual exhaustion, driven by chronic metabolic stress and sustained insulin demand (Khin et al. 2023). Under this metabolic duress, NRF2 induces several cytoprotective and metabolic downstream targets as previously mentioned (Figure 2). In β‐cells, this cascade reduces oxidative stress and preserves insulin secretion machinery. Furthermore, the activation of NRF2 is proposed to contribute to β‐cell reactivation by restoring redox balance and protecting β‐cell mass. In turn, transcriptional identity is reactivated, aiding in the restoration of mature β‐cell phenotypes and enhanced proliferation pathways. Improved cross talk within autophagy and mitophagy pathways help maintain organelle homeostasis and increase β‐cell‐specific activation of NRF2. This increases β‐cell proliferation and mass, improving overall glucose tolerance (Baumel‐Alterzon et al. 2022), protecting against glucolipotoxicity, permitting steadier glucose‐stimulated insulin secretion (Schultheis et al. 2019), and initiating additional self‐repair mechanisms through NRF2‐mediated antioxidant pathways (Abebe et al. 2017).

NRF2 activators, like bardoloxone methyl or sulforaphane, are being studied to investigate their exact antioxidant potential and their role in enhancing β‐cell survival and phenotypes, particularly in cases of early T2D or islet transplantation contexts (Baumel‐Alterzon et al. 2022). This encourages further investigations on the plant species in this review as NRF2 activators and further validates why targeting NRF2 is a strong therapeutic strategy toward ameliorating the progression of T2D.

### 5.3 Mitochondrial Dysfunction and Cell Death in Diabetes

Mitochondrial apoptosis represents a central mechanistic link between oxidative stress and β‐cell loss in diabetes yet remains under‐integrated in many nutraceutical studies. This process is dependent on mitochondrial outer membrane permeabilization (MOMP), which is a tightly regulated event regulated by the Bcl‐2 family of proteins. Under glucotoxic and lipotoxic conditions, increased ROS and metabolic stress induce BH3‐only proteins, which activate proapoptotic effectors such as BAX and BAK. Their oligomerization within the mitochondrial outer membrane facilitates the release of cytochrome *c* into the cytosol, representing the point‐of‐no‐return in intrinsic apoptosis (Volpe et al. 2018; Hohorst et al. 2025). Once released, cytochrome *c* binds apoptotic protease‐activating factor‐1 (Apaf‐1), promoting apoptosome formation and activation of initiator caspase‐9, which subsequently activates executioner caspases such as caspase‐3 and ‐7. This cascade drives the proteolytic dismantling of the cell, resulting in β‐cell loss and impaired insulin secretion. Notably, the balance between antiapoptotic proteins (e.g., Bcl‐2 and Bcl‐xL) and proapoptotic proteins (BAX, BAK) determines cellular susceptibility to apoptosis, (Sahoo et al. 2023) positioning this regulatory axis as a critical control point in diabetes‐associated mitochondrial dysfunction.

In parallel, ROS enhances death receptor signaling, notably via Fas and tumor necrosis factor receptor‐1, leading to caspase‐8 activation through the extrinsic pathway (You et al. 2022) to activate caspase‐3. Collectively, ROS‐driven dysregulation of mitochondrial integrity and cell death is a key contributor to diabetes‐associated tissue damage (Samadder et al. 2013; Dinić et al. 2022) highlighting mitochondrial‐sensitive apoptotic checkpoints as potential therapeutic targets.

Rooibos‐derived extracts and bioactive constituents have demonstrated antiapoptotic effects in diabetogenic models. Unfermented Rooibos extracts suppress apoptosis in hepatic cells by inhibiting the activity of key initiator and executioner caspases, including caspase‐8, caspase‐9, and caspase‐3 (Abdul and Marnewick 2022). Similarly, a pharmaceutical‐grade Rooibos extract enriched with aspalathin confers protection to pancreatic β‐cells under diabetogenic stress through downregulation of proapoptotic genes associated with endoplasmic reticulum stress and redox imbalance (Moens et al. 2022). Although data on individual Rooibos‐derived metabolites remain limited, phenylpyruvic acid‐2‐*O*‐β‐d‐glucoside has been shown to mitigate high glucose‐induced cardiomyocyte apoptosis (Dludla et al. 2020) by restoring the Bcl‐2/Bax balance and reducing caspase‐3/7 activity (Dludla et al. 2020), whereas aspalathin decreased the frequency of apoptotic INS1E β cells, these effects were linked to repression of thioredoxin interacting protein and DNA damage‐inducible transcript 3 (Moens et al. 2020), both of which are proapoptotic genes induced under glucotoxic conditions and closely linked to mitochondrial and endoplasmic reticulum stress pathways (Chen et al. 2008; Laybutt et al. 2007). Collectively, these findings position Rooibos as a modulator of intrinsic and extrinsic apoptotic signaling under diabetic‐associated mitochondrial stress. From a mechanistic perspective, the key question is whether plant‐derived polyphenols can modulate these apoptotic checkpoints rather than acting as general antioxidants. Emerging evidence suggests that Rooibos‐derived compounds exert antiapoptotic effects upstream and downstream of MOMP. However, these findings remain fragmented, with most studies focusing on endpoint apoptosis markers rather than systematically interrogating the full mitochondrial apoptotic cascade.

The impact of whole extracts of Honeybush and Bush tea on diabetes‐associated apoptosis remains poorly characterized, representing a clear gap in the literature. A study by Chellan and colleagues showed that unfermented Honeybush tea protected pancreatic β‐cells of diabetic Wistar rats by promoting glucose tolerance (Chellan et al. 2014); however, no molecular mechanism was deduced. Mangiferin, a major xanthone in Honeybush, has been shown to attenuate mitochondrial‐dependent apoptosis in renal tissue of diabetic rats by improving the MMP, and reducing the expression of cytosolic cytochrome *c*, caspase‐9, and caspase‐3 (Pal et al. 2014), equivalent evidence in pancreatic β‐cells is lacking and largely confined to cytoprotective effects (Liu et al. 2025) rather than mechanisms of apoptosis inhibition. For Bush tea, existing studies, with only general toxicity assessments are described (Chellan et al. 2012), with minimal investigation into apoptosis‐specific or mitochondrial mechanisms under diabetogenic conditions.

Comparatively, Rooibos has the most coherent evidence base linking its bioactive constituents to mitochondrial apoptosis, with studies demonstrating suppression of both initiator and executioner caspases, as well as modulation of ER stress‐associated apoptotic signaling. In contrast, mechanistic evidence for Honeybush remains largely centered on mangiferin, which has been shown to stabilize MMP and reduce cytochrome *c* release in non‐pancreatic tissues. Direct interrogation of Bcl‐2 family dynamics and caspase activation in β‐cell models is notably lacking. For Bush tea, apoptosis‐related mechanisms remain almost entirely unexplored, representing a clear gap in the literature.

A major limitation across the current literature is the reliance on indirect or partial markers of apoptosis, without comprehensive mapping of the mitochondrial apoptotic pathway. Few studies simultaneously assess MOMP, Bcl‐2 family regulation, cytochrome *c* release, and downstream caspase activation within the same experimental system. This limits the ability to conclusively determine whether these phytochemicals act as true modulators of mitochondrial apoptosis or primarily as upstream regulators of oxidative stress. Future studies should adopt integrated mechanistic frameworks to resolve these interactions. Addressing these gaps will be essential to establish whether South African herbal teas can meaningfully preserve β‐cell mass through targeted modulation of mitochondrial apoptosis, rather than through indirect antioxidant effects alone.

### 5.4 Mitochondrial Dysfunction and Inflammation in Diabetes

**FIGURE 3:**
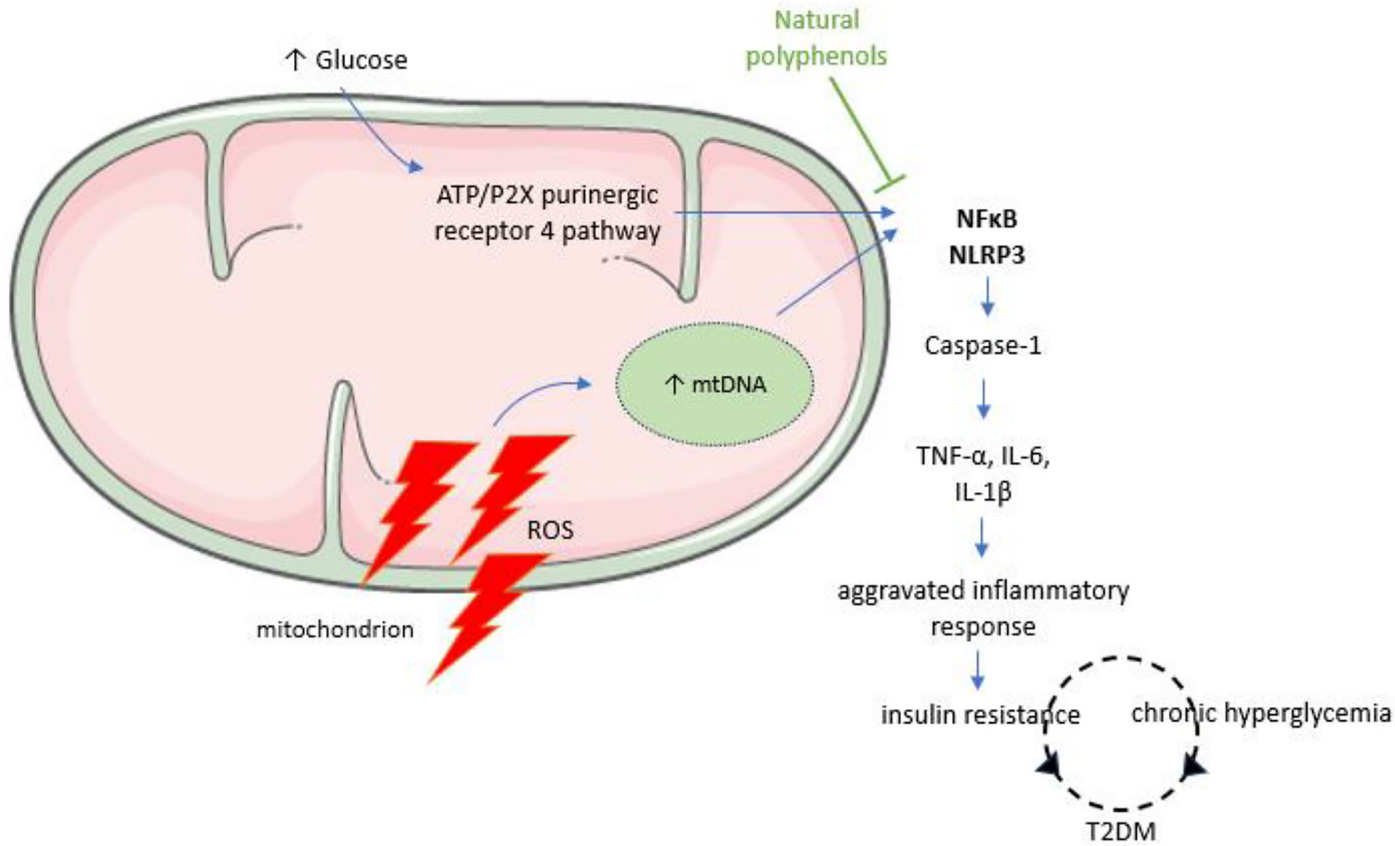
Abnormal glucose and/or dysfunctional mitochondria lead to ROS accumulation, which causes mitochondria to release a large amount of mitochondrial DNA (mtDNA). The increased mtDNA induces nuclear factor κB (NFκB) activity, leading to expression of NLRP3 inflammasome and caspase‐1. This causes an upregulation in inflammatory cytokines that cause an aggravated inflammatory response associated with insulin resistance, encouraging elevated glucotoxicity and the onset of diabetes. Natural polyphenols like rutin and mangiferin suppress activation of NLPR3 and related caspases. IL‐1β, interleukin‐1β; IL‐6, interleukin‐6; ROS, reactive oxygen species; T2DM, Type 2 diabetes mellitus; TNF‐α, tumor necrosis factor‐alpha.

A deregulated and unresolved inflammatory response has been identified as a driver of diabetes in MetS (Tsalamandris et al. 2019). Accumulating evidence has linked mitochondrial dysfunction to inflammation as a central contributor to insulin resistance and pancreatic islet β‐cell failure observed in Type 2 diabetes (Khodabandehloo et al. 2016; Keane et al. 2015). Mitochondria are mediators of the pro‐inflammatory status, by modulating innate immunity through redox‐sensitive inflammatory pathways or direct activation of a group of protein complexes known as inflammasomes. The nucleotide‐binding and oligomerization domain‐like receptor family pyrin domain‐containing 3 (NLRP3) is the best‐characterized inflammasome, which can be activated by damage‐associated molecular patterns (DAMPs) attributed to dysfunctional mitochondria and ROS production (Lu et al. 2023). Damaged mitochondria release a large amount of mtDNA, which activates the NLRP3 inflammasome. The stimulation of NLRP3 results in the activation of caspase‐1 and subsequent maturation of the inactive precursor of interleukin (IL)‐1β, promoting insulin resistance (Strowig et al. 2012), whereas a systematic review and meta‐analysis by Alfadul et al. (2022) showed that IL‐1β prevents proper pancreatic islet function and leads to cell death (Figure 3).

Furthermore, aberrant glucose metabolism plays an important role in the initiation of chronic inflammation through activation and regulation of NLRP3 inflammasome via the ATP/P2X purinergic receptor 4 pathway as well as inducing and promoting the expression of thioredoxin interaction protein, an important cofactor of the NLRP3 inflammasome (Wang et al. 2020) (Figure 3). These pathways may work synergistically to activate inflammatory cytokines, leading to the exaggerated inflammatory response often observed in diabetic patients (Keane et al. 2015; Khodabandehloo et al. 2016).

Natural polyphenols could target the inflammatory pathway. Rutin, a plant pigment, has been spotlighted for its anti‐inflammatory nature and ability to improve metabolic function (Muvhulawa et al. 2022). When administered orally, rutin undergoes metabolic degradation within the gastrointestinal tract to produce metabolites, such as isoquercetin, quercetin, and other polyphenolic derivatives. This facilitates the bioavailability and subsequent biological activity of these metabolites. As one of the major polyphenolic constituents of Rooibos, rutin plays a pivotal role in enhancing the health‐promoting properties of this herbal tea and enriches its therapeutic profile. Targeting the NLRP3 inflammasome presents an opportunity for reducing inflammation in the diabetic condition (Figure 3).

NLRP3 inflammasomes require a priming step in which the inflammasome components are upregulated. The nuclear factor κB (NFκB) is responsible for the expression of NLRP3 and caspase‐1 (Bauernfeind et al. 2009). MetS is aggravated by NFκB augmentation of inflammatory cytokines, such as tumor necrosis factor‐alpha (TNF‐α), interleukin (IL)‐6, and IL‐1β (Piatkowska‐Chmiel et al. 2021) (Figure 3). An in vitro study shows the flavonoid downregulation of LPS‐induced NFκB by reducing pro‐inflammatory markers, such as TNF‐α, inducible nitric oxide synthase (iNOS), and IL‐6 (Liu et al. 2019). Comparatively, rutin safeguards against inflammatory responses by decreasing NLRP3, IL‐1α, and purinergic P2X7 ionotropic receptor (P2X7r) in HSC‐T6 cells or primary rat hepatocytes (Hou et al. 2020). Essential oils from Bush tea can counteract the 5‐lipoxygenase enzyme's activity, suggesting an anti‐inflammatory potential (Padayachee 2011). In contrast, the methanol extracts showed no significant anti‐inflammatory effects; however, their effects were potentiated when blended with other teas. Mangiferin, in high glucose endothelial cells, reduced IL‐1β secretion and NLRP3 expression (Song et al. 2015). In addition, mangiferin exerted a protective role in acute liver injury promoted by LPS and d‐galactosamine (d‐GalN) exposure by suppressing the expression of iNOS along with related NLRP3, ASC, and caspase‐1 (p10) expression and, consequently, the production of IL‐1β (Pan et al. 2016).

The research findings underscore the significance of exploring the benefits of Bush tea, particularly when combined with other teas and tisanes, to augment its health‐promoting properties potentially. The evidence strongly advocates for the protective role of flavonoids present in Rooibos and Honeybush, particularly in shielding against the inflammasome pathway in models that mimic MetS. This suggests a promising area for future research to further elucidate the therapeutic potentials of teas in metabolic health management.

From Table 3, the interconnected cellular pathways highlight the potential of South African herbal teas and their bioactive compounds in modulating cellular processes related to oxidative stress, inflammation, and glucose metabolism, offering therapeutic prospects for managing associated MetS pathologies.

**TABLE 3:** A summary of the key bioactive compounds and how they form part of the interconnected cellular pathways.

	Most potent for	Key bioactive compound(s)	Key pathways	References
Rooibos	β‐Cell regeneration, glucose control	Aspalathin	Activation of NRF2/Keap1, AMPK, ARE and or PI3K/Akt, and GLUT4 transporters. Inhibition of NF‐κB, reduction of pro‐inflammatory cytokines	Muller et al. (2012)
Honeybush	Mitochondrial protection, lipid/glucose metabolism	Mangiferin, hesperidin	Activation of NRF2/Keap1, AMPK, SIRT1. Inhibition of NF‐κB, reduction of pro‐inflammatory cytokines	Jayasuriya et al. (2024), Pal et al. (2014), Chao‐Qiang et al. (2017)
Bush tea	Anti‐inflammatory, vascular health	Quercetin, apigenin, and rutin	NF‐κB, Akt/eNOS, AGE‐RAGE. Limited studies on mechanisms, requiring further elucidation	Malongane et al. (2022)

Abbreviations: ARE, antioxidant‐response‐element; NRF2/Keap1, nuclear factor erythroid 2/Kelch‐like ECH‐associated protein 1; SIRT1, sirtuin 1.

Across the oxidative stress and inflammatory literature, a consistent pattern emerges in that Rooibos and Honeybush repeatedly affect NRF2‐linked antioxidant signaling and NF‐κB/NLRP3‐associated inflammatory pathways, whereas evidence for Bush tea remains preliminary and largely indirect. Rooibos appears particularly well supported for enhancing endogenous antioxidant enzymes and suppressing glucotoxic oxidative injury, whereas Honeybush shows notable activity through mangiferin and related compounds in inflammation models. Bush tea contains promising flavonoids with recognized anti‐inflammatory properties, but direct evidence linking these compounds to mitochondrial redox control in diabetes remains limited. Therefore, the current literature supports a shared mechanistic framework across these teas but also clearly indicates that the translational maturity of the evidence differs by plant species and pathway.

## 6 Considerations

Natural products, particularly those of plant origin, are a source for discovering promising lead candidates for upcoming drug development programs. The common thread for these SA herbal teas as lead candidates includes their extensive polyphenolic profiles and resultant antioxidant activity, in addition to their various other bioactive roles. The absence of alkaloids and low tannin content in these herbal teas, together with the bulk of studies advocating their safety, and only a few contradictory studies suggesting possible damaging effects (Reddy et al. 2023), make these herbal teas an attractive focal point when investigating the therapeutic potential of polyphenols as diabetic interventions.

However, it is still important to consider that “natural” does not guarantee “safety” as several undesirable properties of polyphenols have been disclosed. This includes genotoxicity, thyroid damage, hormone disorders, and interactions with other drugs. Additionally, bioactivities vary among polyphenolic constituents and food sources, with no specific food or polyphenol proven to better all phenotypes of diabetes. Antioxidant efficiency of phenols and other molecules is also strongly dependent on its ability to reach specific targets at the required concentration. It is also important to note that compounds do not work in isolation and synergize cellular antioxidant mechanisms and metabolic machinery instead. The interplay between phytochemical composition, processing, and preparation strongly influences the bioactive profile and functional potential of Rooibos and Honeybush. From a food science perspective, these factors are central to the development of functional beverages and nutraceutical products with consistent quality, efficacy, and consumer acceptability. Therefore, future studies should also focus on the potential cytotoxicity of polyphenols, dosage considerations, as well as drug‐drug interactions and their synergistic effects, to determine their true safety and efficacy profiles (Liu et al. 2019). Moreover, future research also prioritizes optimization of processing technologies, enhancement of bioavailability, and validation through human clinical studies to support evidence‐based functional food applications (Abd Alelah et al. 2026).

Given that mitochondrial impairment and excessive ROS production are central features of insulin resistance and β‐cell dysfunction, the incorporation of these bioactive compounds into palatable products that can be consumed daily offers a practical strategy to support mitochondrial resilience and metabolic health. The translation of these mechanistic benefits into effective functional products requires careful consideration of formulation parameters. The stability of mitochondria‐relevant bioactives during processing and storage is critical, as degradation of compounds may reduce their capacity to modulate oxidative stress and downstream signaling pathways implicated in glucose homeostasis. Conversely, the enhancement of relatively stable compounds may provide more consistent bioactivity in formulated products.

Processing conditions, optimization of extraction methods, and encapsulation approaches may enhance the preservation and bioavailability of these compounds, potentially improving their capacity to exert protective effects on mitochondrial function in vivo. Interactions within complex food matrices may influence the delivery and efficacy of these bioactives, further emphasizing the need for formulation‐driven optimization.

Considering oxidative stress alone, the speculated mechanism of action of the herbal teas for antioxidant efficiency of polyphenols is through proper regulation of the oxidoreductase enzyme system, the induction of antioxidant enzymes, inhibition of pro‐oxidant enzymes, and restoration of mitochondrial function, suggest that these teas hold strong potential to alleviate the phenotypes and pathogenesis of diabetes (Liu et al. 2019). Bearing in mind that NRF2 shows promise in the regulation of β‐cell mass, it is an attractive therapeutic target for expanding and protecting β‐cell mass in diabetes. However, this needs to be explored further, to better understand whether transient or sustained NRF2 activation would be better for β‐cell reactivation and whether NRF2 activation can be selectively targeted to β‐cells without compromising other tissues.

## 7 Conclusion and Perspectives

Diabetic patients are becoming increasingly dependent on polypharmacy, escalating healthcare costs, treatment complexity, and non‐compliance (Saeed et al. 2024; Dobrică et al. 2019). These challenges highlight the necessity for preventative and adjunct interventions that are not only biologically effective but also socioeconomically sustainable. Nutraceutical strategies that target metabolic dysregulation, oxidative stress, and mitochondrial dysfunction offer an exciting avenue to complement conventional pharmacotherapy and reduce long‐term healthcare pressures.

Although Rooibos, Honeybush, and Bush tea demonstrate promising bioactivity in preclinical systems, the current evidence base is largely restricted to in vitro and animal studies, with a paucity of well‐controlled human clinical trials. Consequently, their potential nutraceutical value should be regarded as preliminary, and any efficacy claims must remain proportionate to the available level of evidence.

To advance translational relevance, future research should prioritize rigorous clinical validation through human intervention studies assessing outcomes such as glycemic control, oxidative stress, inflammation, and mitochondrial function using validated biomarkers. In parallel, phytochemical standardization is essential to ensure consistency in bioactive composition, accounting for variability introduced by plant processing, fermentation, preparation methods, and bioavailability. Such standardization would enhance reproducibility and enable meaningful cross‐study comparisons.

From a food science and translational development perspective, further work is also required to optimize formulation strategies that enhance stability, bioavailability, and consumer acceptability of these teas as functional products. This includes systematic evaluation of dose–response relationships, safety profiles, food matrix interactions, and the influence of delivery systems such as encapsulation on phytochemical integrity and biological activity. Collectively, these steps are necessary to bridge the gap between promising preclinical findings and the development of evidence‐based functional foods and nutraceuticals with genuine clinical and commercial applicability.

## Author Contributions

**Shanika Reddy**: conceptualization, writing – original draft, writing – review and editing. **Naeem Sheik Abdul**: conceptualization, writing – original draft, writing – review and editing, supervision. **Taskeen Fathima Docrat**: writing – original draft, writing – review and editing, conceptualization. **Jeanine Marnewick**: writing – review and editing, supervision.

## Funding

This work was supported by the National Research Foundation, South Africa (Thuthuka Grant TTK23032186697 to NSA and Postdoctoral fellowship PSTD2204203929 to SR).

## Conflicts of Interest

The authors declare no conflicts of interest.
